# A three-stage sequential surgical approach to a more efficient management of clinical stage 4 diabetic foot ulcers

**DOI:** 10.3389/fsurg.2025.1696424

**Published:** 2025-11-27

**Authors:** Fang Zhang, Yu Guo, Wenduo Zhang, Ilaria Dal Prà, Wei Chen, Xiaojin Mo, Hehua Song, Anna Chiarini, Jinpiao Yang, Kaiyu Nie, Zairong Wei, Shusen Chang

**Affiliations:** 1 Department of Burns and Plastic Surgery, Affiliated Hospital of Zunyi Medical University, Zunyi, Guizhou, China; 2 The Collaborative Innovation Center of Tissue Damage Repair and Regeneration Medicine, Zunyi Medical University, Zunyi, Guizhou, China; 3 Department of Surgery, Dentistry, Pediatrics & Gynecology, University of Verona Medical School, Verona, Venetia, Italy; 4 Organ Transplantation Center, Affiliated Hospital of Zunyi Medical University, Zunyi, Guizhou, China

**Keywords:** diabetic foot, ulcers, bone cement, angioplasty, tibial cortex transverse transport

## Abstract

**Background:**

It has been a great challenge to treat clinical stage 4 Diabetic foot ulcers (DFUs) due to high rates of major amputations and prolonged healing time. This study aimed to assess the effectiveness of a three-stage sequential surgical approach, which based on the Integrated Surgery Wound Treatment (ISWT) mode, to manage clinical stage 4 DFUs and compare the benefit of incorporating tibial cortex transverse transport (TTT) surgery at stage 3 treatment.

**Methods:**

Twenty-three patients with clinical stage 4 DFUs aged 45–75 years treated between January 2022 and February 2023 were retrospectively analyzed. Eleven patients (Group A) received wound debridement, antibiotic-loaded bone cement (ALBC) at stage 1 treatment, percutaneous transluminal angioplasty (PTA), wound debridement, and ALBC at stage 2 treatment, and skin grafting with TTT at stage 3 treatment, while twelve patients (Group B) received the same treatment without TTT. Assessed clinical outcomes included length of hospital stay, ulcer healing duration, ulcer recurrence rate, reintervention (re)-PTA rate, amputation rate, mortality rate, visual analog scale (VAS) scores, ankle-brachial index (ABI), and two-point discrimination (2-PD) ability. The computed tomographic angiography (CTA) was used to evaluate vascular hyperplasia.

**Results:**

Group A showed no occurrences of re-PTA (*P* = 0.037) and similar ulcer healing times (*P* = 0.975) compared to Group B. Ulcer outcome, amputation, and mortality rate were also alike in the two groups (*P* > 0.05). One year after surgery, Group A demonstrated improvement in VAS scores, ABI, and 2-PD, while Group B showed no significant changes. Additionally, Group A exhibited enhanced lower limb artery characteristics compared to Group B.

**Conclusion:**

The sequential three-stage approach based on the ISWT mode effectively manages clinical stage 4 DFUs. Incorporating TTT surgery at stage 3 extends the benefits of PTA surgery.

## 1 Introduction

Diabetic foot ulcers (DFUs) were usually characterized with varying degrees of peripheral neuropathy, infection, and ischemia (1). Moreover, diabetes contributes to the onset of peripheral arterial disease (PAD) through the interplay of hemodynamic, neurohormonal, and metabolic factors. PAD potentially results in severe infections and amputations and significantly contributes to disability and mortality in patients with diabetes (2). Of note, the 5-year mortality after amputation in these patients is exceedingly high, ranging from 39% to 80% (3). In addition to the financial burden inherent to the treatments, patients with DFUs experience a significant decline in the quality of their life (4).

When treating DFUs, it is essential to strive to avoid major amputations (5). Characterized with deep ulcer and extensive gangrene involving at least forefoot and/or midfoot which result in high rates of major amputations and prolonged wound healing times, it has been a great challenge for the treatment of clinical stage 4 DFUs graded according to the Society for Vascular Surgery (SVS) Wound, Ischemia, and foot Infection (WIfI) classification system (6). The current therapies focus on the timing, choice of modality, and success of revascularization, with ulcer healing often considered as a secondary criterion (7). Marston et al. (8) found that the ulcer healing rate after revascularization was approximately 25% within six months, while at one year, the healing rate ranged from 46% to 91%. Despite these efforts, the overall ulcer healing rate remained modest, and the healing time was lengthy. Percutaneous transluminal angioplasty (PTA), which is demonstrably workable and technically effective, is now widely used in patients with DFUs and critical limb ischemia (CLI), especially in those who are not eligible for open surgery or who suffer from multiple complications (7, 9). However, restenosis or occlusions of the blood vessels after PTA are still common events requiring reintervention (re)-PTA. Within one year, the rates of ulcer recurrence and major amputation have remained high, regardless of the need for re-PTA (10).

Free tissue transfer combined with revascularization promotes healing by effectively covering various defects with well-perfused tissue, providing and augmenting blood flow to ischemic areas, and easing venous drainage in districts with venous insufficiency. Currently, free tissue transfer is considered as one of the best treatments for DFUs (11, 12). When contemplating limb salvage through free tissue transfer, the limb vascularity becomes a crucial consideration. Therefore, perfecting limb blood flow through PTA is essential to achieve a successful free tissue coverage and to maximize limb salvage rates (13). However, opening sub-popliteal arteries for revascularization using PTA, particularly those in the foot and ankle, presents significant technical challenges in clinical stage 4 DFUs patients. Even if PTA is successful, the extensive arterial wall damage and calcification make the microscopic anastomoses difficult to set up. Arterial anastomoses carry an elevated risk of failure and are associated with a high incidence of complications, including vascular crises, partial or complete necrosis of the flap, and delayed healing (12, 14). Therefore, exploring alternative, dependable, and effective methods with a high long-term limb salvage rate is crucial.

In recent years, the Integrated Surgery Wound Treatment (ISWT) model has been introduced for treating DFUs, as it allows the implementation of various DFUs reconstruction plans (15). The present study reports our experience and benefits in a three-stage sequential treatment plan based on the ISWT mode in clinical stage 4 DFUs. The first stage involves debridement and the application of antibiotic-loaded bone cement (ALBC), which aims at eliminating necrotic tissue and effectively controlling wound infection. During the second stage, we perform PTA to open the blood vessels, followed by debridement and the application of ALBC, whose purpose is to increase blood flow in the affected limb, improve the blood circulation of the wound bed, and at the same time, eliminate the infection through the application of ALBC and promote the formation of the induced membrane, thus creating conditions for the subsequent repair of the wound. At the third stage, we employ tibial cortex transverse transport (TTT) combined with skin grafting for tissue repair as TTT surgery can improve the microcirculation of the lower limbs through the stress-tension mechanism (15), enhance the blood supply to the wound bed so that promote the survival of the skin grafts. For comparison purposes, we conducted only skin grafting at the third stage. Our findings suggest that the three-stage sequential surgical approach based on the ISWT model can rapidly control infection, accelerate wound healing, and achieve limb salvage. In the third stage, the combination with TTT promotes microcirculation regeneration, enhances sensory function, and decreases the rate of ulcer recurrence. Hence, our approach lessens the need for re-PTA and improves the patient's quality of life.

## 2 Materials and methods

### 2.1 Study design and patient selections

We retrospectively analyzed 23 consecutive cases diagnosed with clinical stage 4 DFUs, who were treated in our institution with debridement and ALBC at stage 1 treatment, PTA and ALBC at stage 2 treatment, and skin grafting combined with or without TTT at stage 3 treatment from January 2022 to February 2023. At stage 3 teatment, 11 cases received TTT treatment (Group A), while 12 cases did not (Group B). All patients in our study had a complete follow-up for at least one year. Informed consent was obtained from all patients. Approval was obtained from the ethics committee of the Affiliated Hospital of Zunyi Medical University, Zunyi, China (Approval No. KLL-2023-623). The study protocol conforms to the ethical principles of the Helsinki Declaration as reflected in *a priori* approval by the Institution's human research committee. A comprehensive review was conducted to extract demographics, characteristics, comorbidities, and clinical outcomes during the study period. All patients showed peripheral neuropathy and moderate to severe limb ischemia, with no associated cardiac, hepatic, or renal dysfunctions (Table 1 and Supplementary Table S1). Following PTA treatment, two sub-popliteal arteries were opened in all patients.

**Table 1:** Characteristics of patients.

Characteristic	Group A	Group B	Statistical value levels	*P*-value
Age (years)	56.4 ± 7.6	56.8 ± 7.5	*t* = −0.149	0.883
Gender				
Male	7 (63.6%)	7 (58.3%)	-	1.000*
Female	4 (36.4%)	5 (41.7%)		
Onset of ulcer (days)	13.6 ± 5.5	13.7 ± 4.6	*t* = −0.057	0.955
Time since diagnosis of type II diabetes (years)	10.4 ± 3.1	10.6 ± 4.0	*t* = −0.145	0.886
Nutritional status(mild/moderate/severe)	2/7/2	2/7/3	-	1.000*
Ulcer area (cm^2^)	89.9 ± 15.7	89.7 ± 26.7	*t* = 0.027	0.979
Location of stenosis/occlusion			-	0.870*
Femoro-popliteal + Infra-popliteal	3	5		
Femoro-popliteal	3	3		
Infra-popliteal	5	4		
HbA1c/%	11.8 ± 1.8	12.5 ± 2.2	*t* = −0.826	0.418
Follow-up time (months)	14.6 ± 2.0	14.8 ± 2.0	*t* = −0.244	0.810

HbA1c, glycated hemoglobin.

* Fisher's exact test.

### 2.2 Surgery procedures

PTA and other surgeries were performed by the same operating surgeons separately in all patients.

#### 2.2.1 Stage 1 operation procedures: debridement and ALBC filling

**Figure 1:**
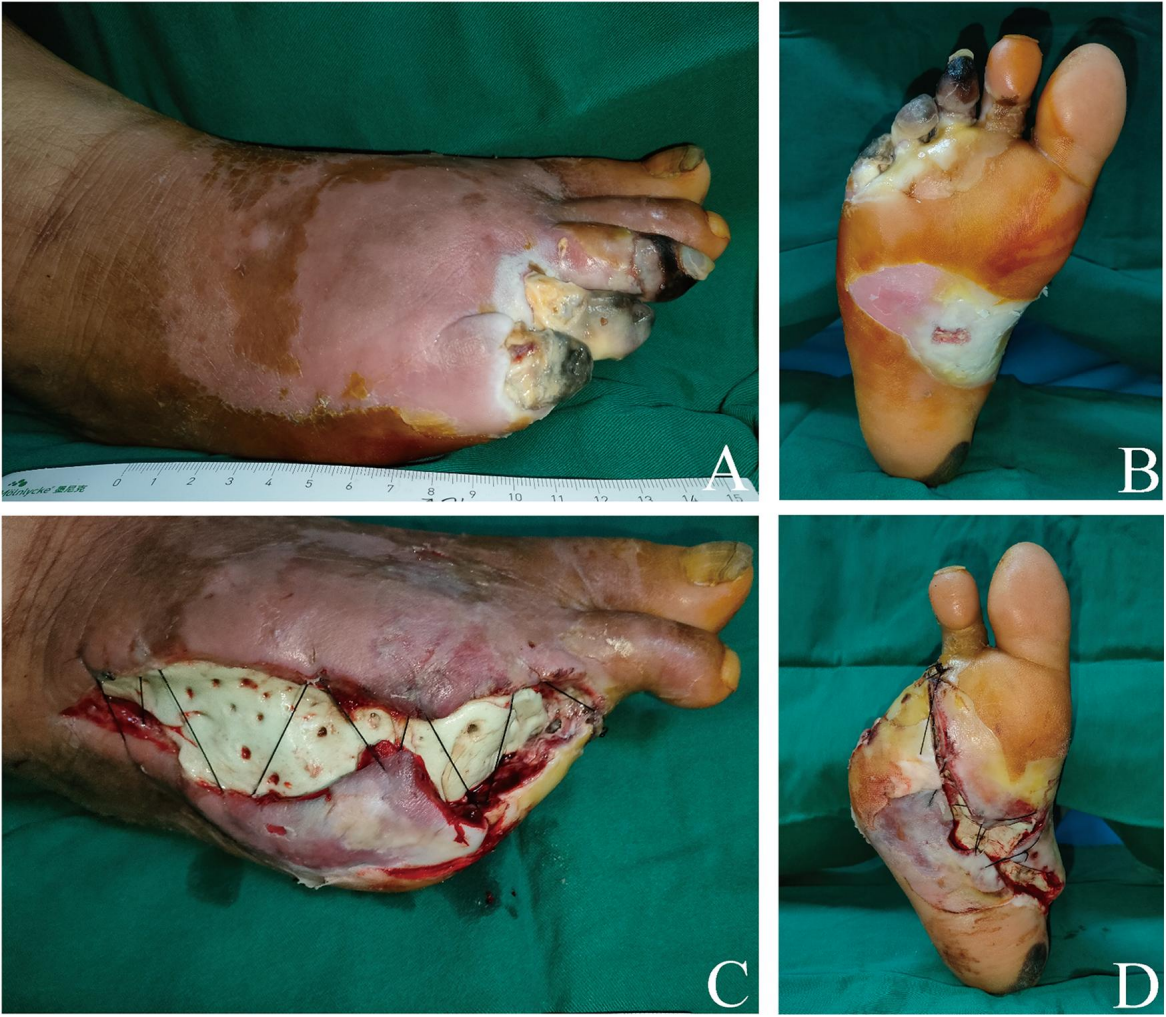
A 51-year-old male with a severe clinical-stage 4 diabetic right foot. The nonviable soft tissues and necrotic toes were debrided, and the defect was filled with ALBC. **(A,B)** The wound before surgical debridement. Dorsal foot aspect **(A)** Plantar foot aspect **(B) (C,D)** Picture of the first dressing after the first-stage surgery. Dorsal foot aspect **(C)** Plantar foot aspect **(D)**

All surgeries were performed under general anesthesia. During the debridement process, we removed necrotic and infected tissue, as well as infected and necrotic bone, until the soft tissue and bones at the edge of the wound were healthy. Thereafter, PALACOS R + G-High-Viscosity Bone Cement with Gentamicin® (Heraeus, Hanau, Germany) was used to cover the wound surface and fill the cavity. Vancomycin (2 g per 40 g mix) was added to the powder before mixing with the liquid. Drainage holes were drilled in the bone cement, which was then secured to the wound using 4# silk sutures. It is essential that the surgeons wait until the bone cement mixture temperature has significantly decreased before covering the wound, thereby avoiding any heat-caused damage to the soft tissue. Sterile dressings were applied and changed every 2–3 days (Figure 1).

Postoperatively, measures were taken to actively manage blood glucose levels, correct any internal environment disorders, and administer cefamandole sodium empirically as the anti-infective treatment (2 g every 8 h for three days). Wound secretion culture and drug sensitivity test were performed on admission, which was the same in stage 2 treatment. Based on the results of wound drug sensitivity testing, adjustments to the type of antibiotic were promptly made. All patients were treated with antibiotic for a total of one week.

#### 2.2.2 Stage 2 operation procedures: PTA and ALBC filling

Approximately two weeks after stage 1 treatment, the patient's blood glucose, nutritional status, and clinical signs and symptoms of infection were effectively controlled. The PTA procedure was performed during this stage.

Before the operation, the patient received a subcutaneous injection of low molecular weight heparin (5,000 IU every 12 h for three days). The surgical procedure was carried out under local anesthesia. The approach for puncture, whether ipsilateral or contralateral, was determined based on the location of the arterial lesions in the lower extremities as detected through the patient's imaging examination. Following a successful puncture, a 6F vascular sheath was inserted, and angiography was performed to reconfirm the location of the occluded artery. Subsequently, intravenous heparin was administered at a dosage of 0.6–0.8 mg/kg, followed by an added dose of 10 mg/h. The occluded artery was navigated using an angiography catheter or support catheter with a 0.035-inch super-smooth or V18 guidewire. Ordinary balloons of varying sizes were then selected to gradually pre-dilate the occluded artery. The baseline time point was considered when the residual stenosis was ≤30% following the completion of surgical predilation. Thereafter, a digital subtraction angiography (DSA) was conducted on the affected limb's artery to evaluate its patency. Once confirmed, a drug-coated balloon for dilation of a proper size was selected, with paclitaxel (at a concentration of 3 µg/mm²) as the coating drug for dilation. The dilation was performed for 180 s at a pressure range of 8–10 atmospheres. After dilation, a second angiography was con-ducted to see the correction of the stenosis. The operation was successful if the second angiography showed a smooth blood flow in the affected area with a <30% residual stenosis. If the residual stenosis was >30% or a flow-limiting dissection occurred, arterial stents would be placed as part of the surgical approach. Notably, no arterial stents were used in this study.

**Figure 2:**
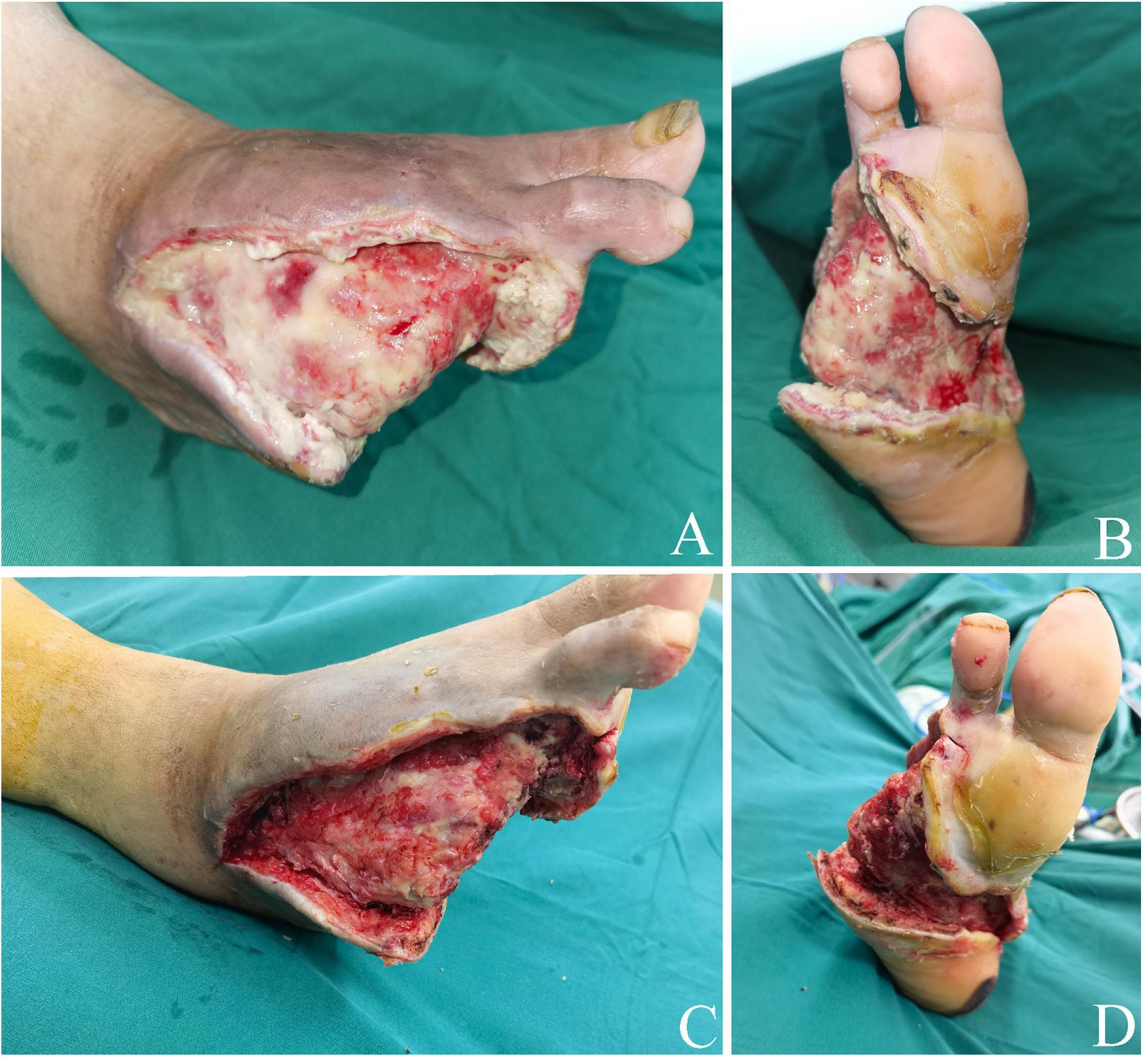
Stage 2 ALBC surgery. **(A,B)** ALBC was removed after PTA surgery. There is still necrotic tissue in the wound. Dorsal foot aspect **(A)** Plantar foot aspect **(B) (C,D)** The nonviable and necrotic soft tissues were debrided. Dorsal foot aspect **(C)** Plantar foot aspect **(D)** ALBC, antibiotic-loaded bone cement; PTA, percutaneous transluminal angioplasty.

Debridement is necessary when there is still necrotic tissue in the wound. After the procedure, the wound will be treated with ALBC, as with the stage 1 surgical approach (Figure 2).

After the operation, the patient received a subcutaneous injection of low molecular weight heparin calcium (5,000 IU, q12 h) for three days and then discharged. Upon discharge, the patient was prescribed oral aspirin (100 mg, qd) and rivaroxaban tablets (2.5 mg, bid) for three months. Furthermore, patients underwent regular evaluations at the outpatient clinic to assess their physical conditions, check blood sugar levels, change wound dressings, and receive symptomatic treatment when necessary.

### 2.2.3 Stage 3 operation procedures: skin grafting combined with or without TTT

The patients were readmitted three weeks after undergoing stage 2 surgery. Stage 3 involved ALBC removal and skin grafting combined with or without TTT. The selection criteria and exclusion criteria for TTT surgery were as follows: selection criteria: after PTA, superficial femoral artery and popliteal artery of lower limbs were unobstructed, and at least one branch of anterior tibial artery, posterior tibial artery and peroneal artery reached the ankle joint plane unobstructed; exclusion criteria: patients with a history of cardiac failure, multiple organ dysfunction, cancer, or renal failure. The procedures were also conducted under general anesthesia with no tourniquet control in the affected limb.

**Figure 3:**
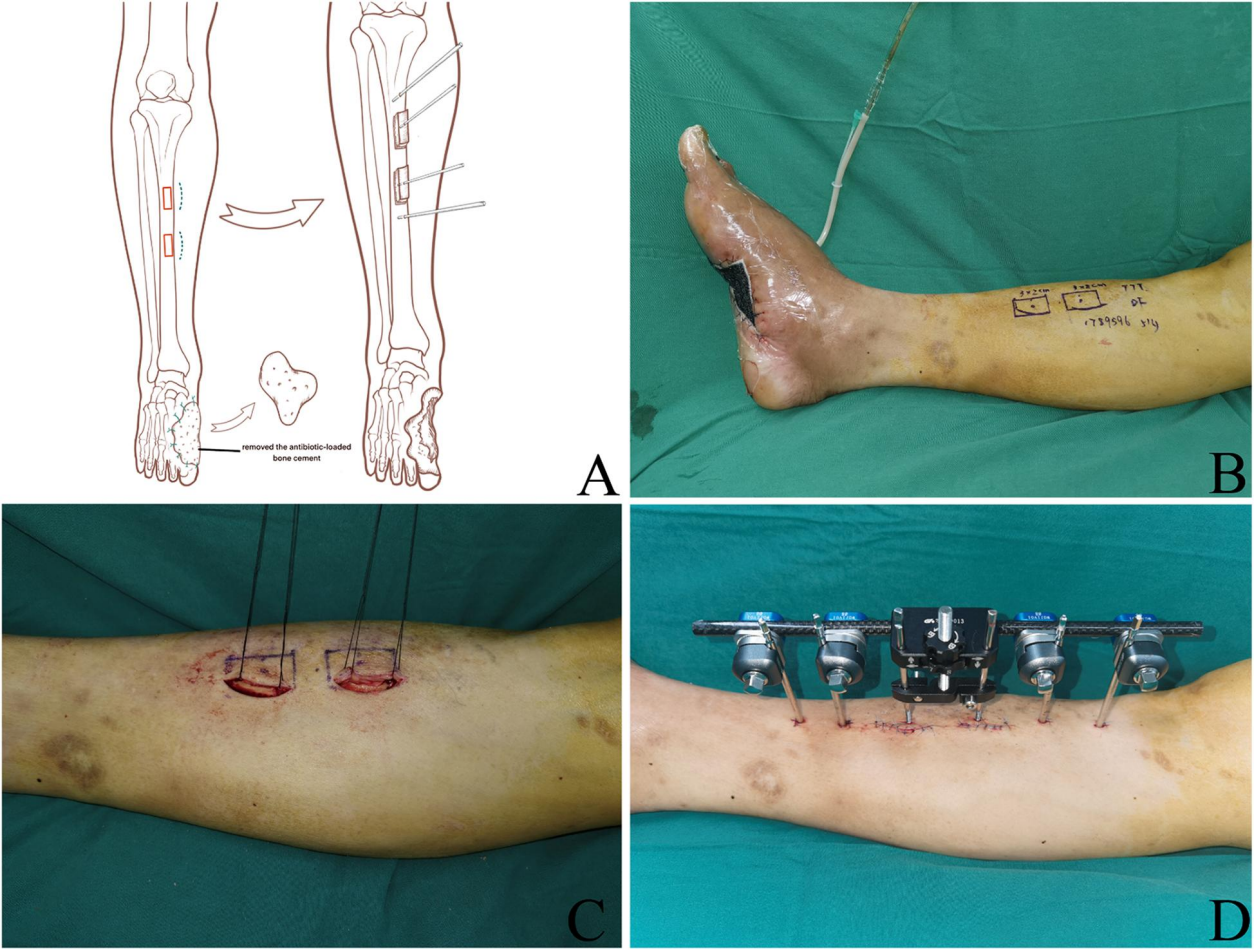
TTT surgical procedures. **(A)** Schematic diagram of a two-segment osteotomy with TTT. **(B)** Intraoperative bone flap design. **(C)** Intraoperative osteotomy. **(D)** The immediate appearance after the TTT procedure completion. TTT, tibial cortex transverse transport.

TTT Surgery: Two 2.0 cm arc-shaped surgical incisions were meticulously made through the skin, reaching the periosteum in the middle-upper section of the tibia. The periosteum was sutured with the skin to prevent separation. After this, the skin and the periosteum were lifted outward, freeing the planned osteotomy site. Two bone blocks measuring 3.0 cm in length and 2.0 cm in width were carefully delimited. The longitudinal cortical bone was bilaterally cut off using a thin osteotome. A 3.0 mm Schanz pin was inserted into each bone cortex, and an external fixation support was also applied. The bone cortices on both sides were transversely cut. Once it was confirmed that the bone cortex osteotomy was complete, the periosteum, subcutaneous tissue, and skin were sutured layer by layer (Figure 3).

**Figure 4:**
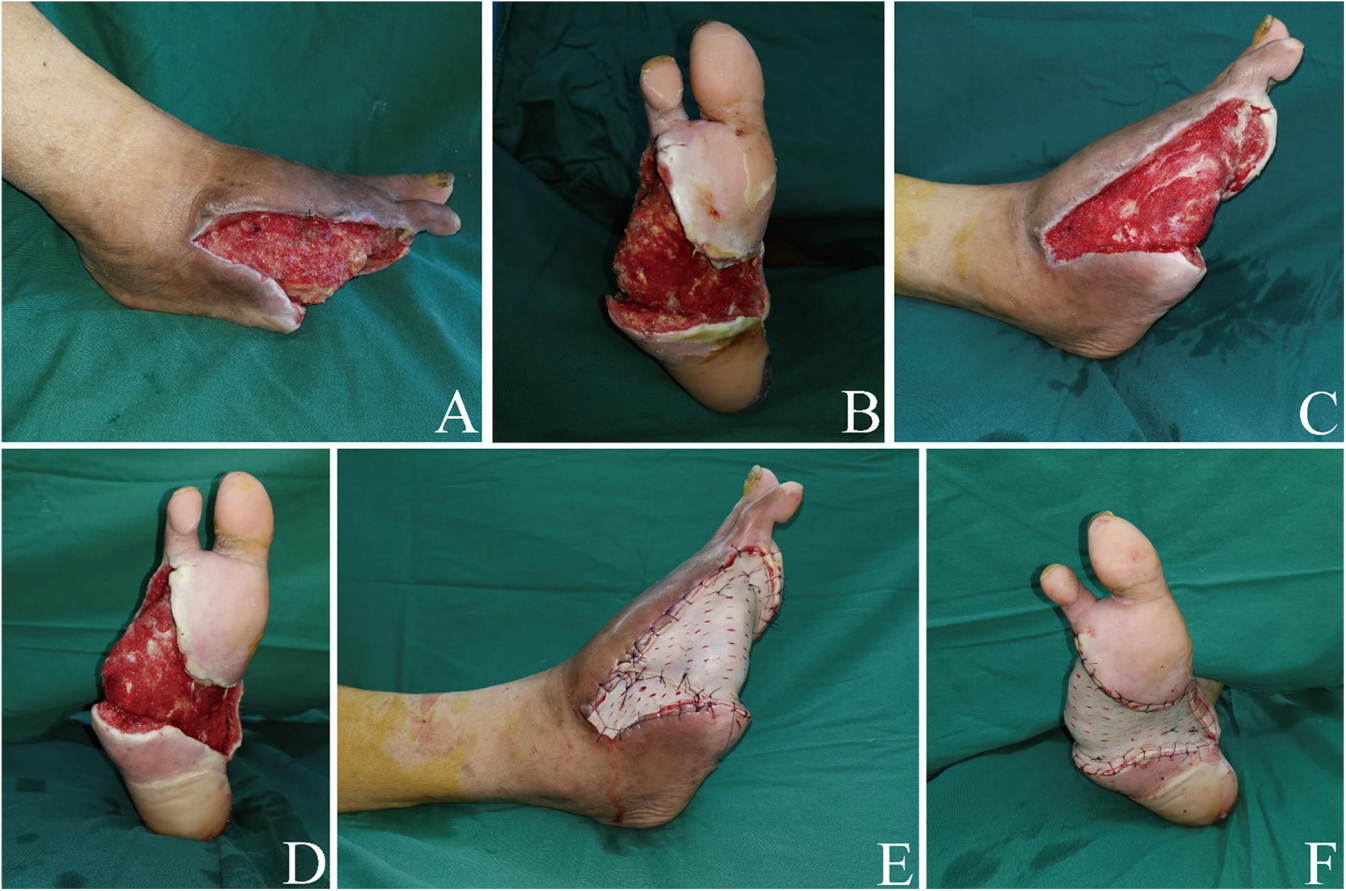
Debridement and skin grafting in the third stage. **(A,B)** The granulation tissue had grown significantly after ALBC's removal. Dorsal foot aspect **(A)** Plantar foot aspect **(B) (C,D)** Wound's appearance post-debridement. Dorsal foot aspect **(C)** Plantar foot aspect **(D) (E,F)** Foot appearance immediately after skin grafting. Dorsal foot aspect **(E)** Plantar foot aspect **(F)** ALBC, antibiotic-loaded bone cement.

Skin grafting: A thin to medium-thickness skin graft was harvested from the thigh and punched for later use. Following debridement, the wound was cleansed with normal saline. The skin graft was then applied to cover the wound and fixed to it (Figure 4). Finally, a negative pressure wound therapy system (VSD Medical Science and Technology Co. Ltd., Wuhan, China) was applied to promote a best healing of the skin graft.

**Figure 5:**
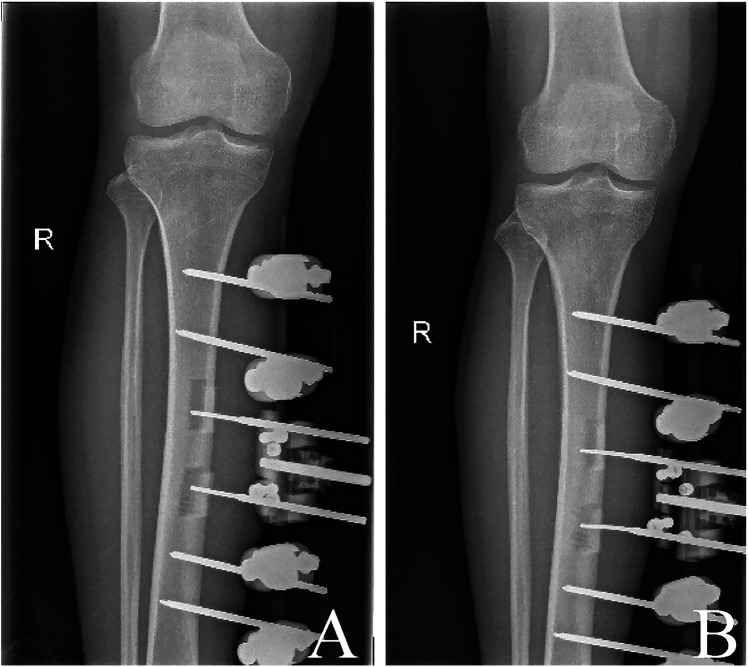
X-ray images. **(A)** 12d radiograph of bone distraction. **(B)** Radiograph on the day of removal of the external fixation frame.

Postoperatively, the patient received treatment including measures to prevent infection and control blood glucose levels. On the fourth day after surgery, bone distraction was started at a rate of 1.0 mm per day. This process typically took four repetitions, with intervals of six hours (Q6 h) between each one. The duration of bone distraction ranged from 12 to 14 days and was adjusted according to skin tension. After keeping the complete distraction for three days, the bone was returned to its original position at the same speed but in the reverse direction. After completing these procedures, x-ray images were reviewed. If the bone fragment had been appropriately repositioned, the external fixator was removed (Figure 5). A brace was recommended to immobilize the area for 4–6 weeks to prevent tibial fractures.

### 2.3 Outcome assessments

During the follow-up, we recorded the length of hospital stay, time for ulcer healing, and rates of ulcer recurrence, re-PTA, amputation, and mortality one year following the last surgical procedure. We compared the changes in visual analog scale (VAS) scores, ankle-brachial index (ABI), and two-point discrimination (2-PD) of the heel in the affected foot of patients before PTA surgery and one year after the last surgical intervention. Computed tomographic angiography (CTA) was performed to evaluate vascular hyperplasia after the last surgical intervention.

### 2.4 Statistical analysis

All statistical analyses were conducted using the SPSS 29.0 software package (Version 29.0; IBM Corp., Armonk, NY, USA). Normally distributed measurements were presented as mean values ± standard deviation (SD). Comparisons between the two groups were conducted using the independent samples t-test. Counts data were expressed as the number of cases and compared using Fisher's exact test. The significance threshold level was set at *P* < 0.05.

## 3 Results

### 3.1 Baseline characteristics of patients

In the present study, 23 patients diagnosed with clinical stage 4 DFUs, including 14 males and 9 females aged between 45 and 75 years (median age, 56.6 ± 4.9 years), met the inclusion criteria. The mean age of patients in groups A and B were 56.4 ± 7.6 years and 56.8 ± 7.5 years, respectively. Group A consisted of 7 males and 4 females, while Group B consisted of 7 males and 5 females. No statistically significant differences (*P* > 0.05) were observed in age, gender, onset of ulcer, ulcer surface area, length of time since type II diabetes diagnosis, nutritional status, location of arterial stenosis or occlusion, HbA1c, and follow-up time between the two groups (Table 1 and Supplementary Table S1).

### 3.2 Outcomes

**Figure 6:**
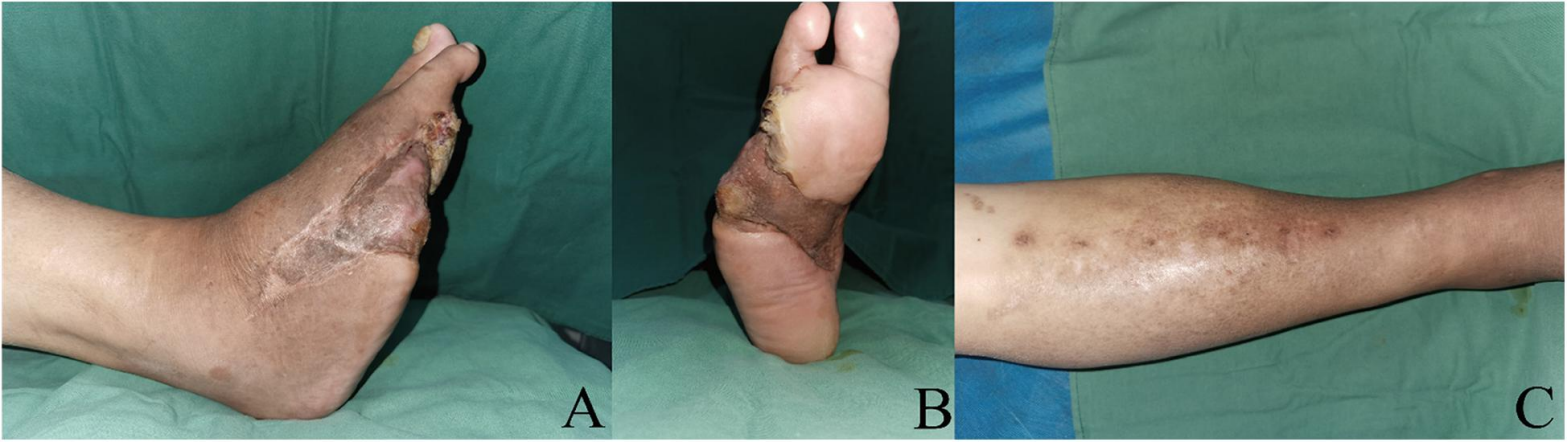
Follow-up at 15 months after the stage 3 procedure. **(A,B)** Appearance of the right foot. Dorsal foot aspect **(A)** Plantar foot aspect **(B) (C)** Appearance of the area after TTT surgery. TTT, tibial cortex transverse transport.

In group A, all patients experienced successful one-stage ulcer healing during the follow-up period, with no ulcer recurrence or complications in the operated areas (Figure 6). The external fixation scaffolds were kept for an average of 32 ± 3.6 days, ranging from 26 to 38 days. No re-PTA was needed in patients in group A (0%) while 5 patients (41.7%) needed re-PTA in group B (*P* = 0.037). The ulcer healing time was similar in both groups, with 56.5 ± 9.5 days in group A and 56.3 ± 8.5 days in group B (*P* = 0.975), respectively. Moreover, there were no statistically significant differences in ulcer healing time, ulcer recurrence, length of stay, major amputation, or death between the two groups (*P* > 0.05) (Table 2).

**Table 2:** Outcomes of patients for each procedure.

Variable	Group A	Group B	Statistical value levels	*P*-value
Healed ulcer time (days)	56.5 ± 9.5	56.3 ± 8.5	*t* = 0.032	0.975
Length of hospital stay (days)	17.5 ± 3.1	16.7 ± 3.3	*t* = 0.528	0.603
re-PTA	0 (0%)	5 (41.7%)	-	0.037*
Major amputation	0 (0%)	1 (8.3%)	-	1.000*
Ulcer recurrence	0 (0%)	3 (25%)	-	0.217*
Death	0 (0%)	2 (16.7%)	-	0.478*

re-PTA, repeated Percutaneous transluminal angioplasty.

* Fisher's exact test.

**Figure 7:**
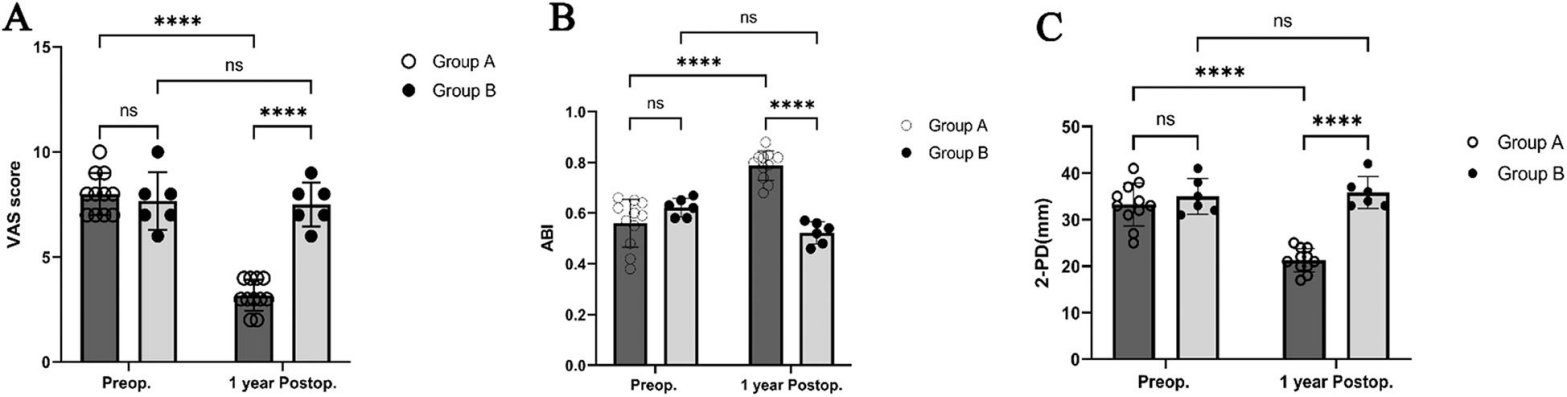
Preoperative and 1-year postoperative VAS scores, ABI, and 2-PD. **(A)** After one year, Group A patients who had had surgery reported significantly less pain than Group B, as shown by their VAS scores. **(B,C)** Significant differences between the two groups also occurred in the one-year postoperative ABI and 2-PD values of the heel (****, *P* < 0.0001). VAS, visual analog scale; ABI, ankle-brachial index; 2-PD, two-point discrimination.

VAS scores, ABI, and 2-PD of the heel were recorded. In group A, significant improvements were seen in VAS scores, ABI, and 2-PD of the heel one year after surgery while group B did not show significant changes in these data at the same deadline (Figure 7). Six cases in group B experienced complications, including one major amputation, three ulcer recurrences, and two deaths, and were therefore excluded from the comparison (Table 2). There was no statistically significant difference in preoperative data between the two groups. However, a statistically significant difference was seen when comparing the 1-year postoperative data (Figure 7).

**Figure 8:**
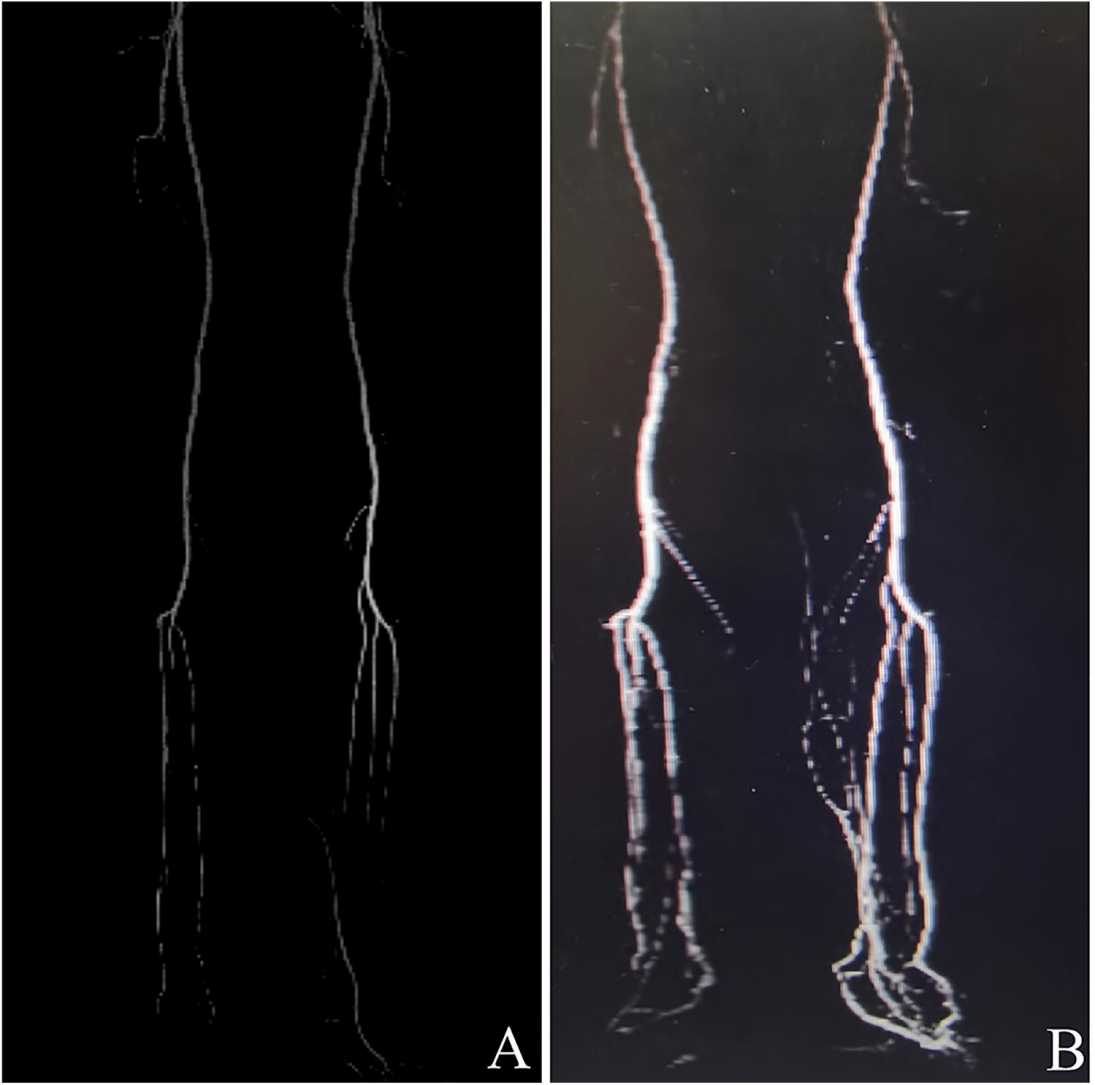
Noninvasive CTA result of a patient who underwent TTT surgery. **(A)** Before PTA surgery, the CTA analysis reveals femoral artery stenosis, infra-popliteal artery stenosis, and occlusion. **(B)** One year after TTT surgery, CTA demonstrates a substantial improvement in vascularization and hemadostenosis at both low extremities, including smoother and thicker arteries, increased vessel numbers, and established collateral circulation. CTA, computed tomographic angiography; TTT, tibial cortex transverse transport; PTA, percutaneous transluminal angioplasty.

In group A, CTA examinations one year after the last operation revealed improvements in the characteristics of the lower limb arteries compared to their preoperative conditions. These improvements included smoother and thicker arteries, an in-creased number of vessels, and the establishment of collateral circulation (Figure 8).

## 4 Discussion

In this study, we assessed the benefits of a three-stage sequential surgical approach in managing clinical stage 4 DFUs. This approach involves debridement and ALBC at stage 1, PTA, debridement, and ALBC at stage 2, and skin grafting combined with or without TTT surgery at stage 3. The therapeutic effects of the combined application of TTT surgery in the third stage were also evaluated.

Local antibiotic therapy has been increasingly used to treat diabetic foot infections (DFIs) (16, 17). However, DFIs often require emergency surgical intervention, and it's can be really difficult to obtaining a preoperative specific antimicrobial profile from bacteria cultures due to the time lag between specimen culture and pathogen identification (18). ALBC is a commonly used antibiotic-loaded material for the treatment of DFIs and can effectively control infection and promote the growth of the granulation tissue. In this study, we added vancomycin to the gentamicin-containing bone cement to provide topical anti-infective coverage against Gram-negative and Gram-positive bacteria.

Previous studies reported that ALBC induced the release of various factors from the local microenvironment, including anti-inflammatory factors and vascular endothelial growth factor (VEGF), which induce the formation of granulation tissue in the wound and promote wound healing (19, 20). Simultaneously, it can also enhance the protein expression of VEGF receptor 2 (VEGF-R2) in the wound marginal tissue, which effectively promotes the proliferation and migration of vascular endothelial cells, contributing to local neo-microvascularization (21). Additionally, ALBC can handle local infections, reduce pathogenic bacteria, and decrease inflammatory reaction by fitting the wound tightly to eliminate dead spaces and slowly releasing antibiotics (22, 23). Thus, we performed a thorough debridement, drainage, and ALBC coverage in the acute phase of DFIs to inhibit bacterial growth and prevent progression of infection and gangrene, thus preventing wound infection from being worsen. This allows clinicians ample time to adjust blood glucose levels, correct internal environment disorders, and address other comorbidities. In particular, it is considered proper that the fasting blood glucose (FBG) values of patient fall between 3.9 and 7.2 mmol/L, postprandial blood glucose (PBG) values range from 6.1 to 11 mmol/L, and GHbA1C be <8.0% in this study. After approximately two weeks, when the patient's systemic status was well corrected, PTA was performed along with further wound debridement and coverage with ALBC. Due to the severe foot infection and necrosis of a part of interstitial tissue in these patients, a second debridement was unavoidable. What should be noted is that due to the partial recanalization of the lower limb vessels following PTA, local blood flow in-creased, and bleeding occurred more frequently after debridement. To avoid removing ALBC for additional hemostatic efforts, debridement and ALBC coverage should be followed after PTA. After three weeks, when the wound infection was effectively con-trolled and granulation tissue grew well, skin grafting for wound repair can be per-formed.

PAD significantly slows DFU healing, which is a direct consequence of the limited supply of oxygen, nutrients, and local factors necessary for the wound-healing process (24). Revascularization surgery aims to restore sufficient blood flow to advance healing (7). In patients with CLI, several studies found a clear trend toward better healing in patients who underwent revascularization compared to those who did not (25, 26). However, the wound healing rate was only around 40%–50% at six months postoperatively and 52%–75% at one year postoperatively in patients who underwent revascularization (27, 28). In addition to wound healing, rest pain is also one of the key factors in performing revascularization (16).

PTA is the preferred revascularization procedure for DFUs with PAD, which can be successfully repeated in most cases (27). All patients in our group underwent vascularization with this modality. Regarding the timing of PTA, it is crucial to consider that patients in acute phase of infection may have more severe symptoms and complications, such as poor glycemic control, high blood viscosity, red blood cell aggregation, and in-creased platelet activity. Performing PTA at this stage may worsen vascular endothelial damage and further activate the platelets and coagulation system, leading to an in-creased incidence of intravascular thrombus formation. In addition, DFUs patients of-ten have risk factors for cardiovascular diseases, such as hypertension, high cholesterol, and obesity. Performing PTA in the acute phase may elevate blood pressure and in-crease the likelihood of cardiovascular events, such as myocardial ischemia and myocardial infarction. Therefore, we recommend that PTA be performed as early as possible after achieving reasonable glycemic control and effective infection treatment. All patients in this study underwent PTA at the second stage.

Microcirculation is often significantly altered in patients with DFUs combined with PAD. Arteriovenous shunts cause a decrease in nutrient blood flow to skin capillaries, resulting in a state of “chronic capillary ischemia” (29). This phenomenon is more pronounced in diabetic patients with PAD than in non-diabetic patients with PAD. It interferes with the healing process. Microcirculation regeneration is essential for slowing ulcer progression, promoting healing, and reducing amputation rates (30). Improving capillary density and promoting collateral vessel formation is a promising approach to stimulate angiogenesis and arteriogenesis and improve ischemic wound healing.

PTA primarily dilates narrowed or occluded blood vessels and restores blood flow patency (31). Although it alleviates the symptoms of lower limb ischemia, it cannot fully restore microcirculation in the affected area or improve peripheral nerve function in such patients. DFUs patients with PAD may experience restenosis or occlusion due to the severity of their vascular lesions and the complexity of the pathological mechanisms involved. These problems typically occur from a few weeks to months after the procedure but can happen in a much shorter period (32). This may be one of the reasons why early treatment with PTA alone showed variable rates of limb salvage and ulcer healing at one year. Previously, we saw significant improvements in VAS scores and ABI in the patients during the early postoperative period. However, the present study found no statistically significant difference between preoperative and 1-year postoperative VAS, ABI, and 2-PD in group B patients. These suggest that PTA surgery may be less effective in keeping vascular patency, improving symptoms, and re-establishing microcirculation in the long term. The comparison of re-PTA between the two groups (0% in Group A vs. 41.7% in Group B, *P* = 0.037) may also explain the above phenomenon. Although there was no statistical difference in comparing ulcer recurrence rates between the two groups, the small sample size of patients in this study may have contributed to this result. Further confirmation is needed by increasing the sample size.

TTT surgery is a well-known treatment for improving microcirculation in the lower extremity (33). Previous studies found that serum VEGF, basic fibroblast growth factor (bFGF), and epidermal growth factor (EGF) are expressed at high levels during the specific phase of bone distraction after tibial osteotomy, which induced the neovascularization, restores blood flow, improved the wound microenvironment, and promoted wound healing (34, 35). Our previous study found that TTT not only induced the regeneration of the lower limb microvascular network but also improved the function of the DFUs peripheral nerves and the quality of wound healing (15). Cellular therapies and biomaterial scaffolds had been investigated to improve the neovascularization and promotes wound healing, it can be speculated that TTT combined with these therapies may achieve better results. However, more investigations are needed to verify this conjecture in the furture. Considering the possible occurrence of vessel restenosis or occlusion several weeks after PTA, we performed TTT surgery three weeks after PTA when there was no stenosis or occlusion of large vessels, the wound infection was wholly controlled, and there was a good growth of granulation tissue in the wound, which is more conducive to the safety, and successful recovery of the patient after surgery.

Earlier TTT surgical protocols were prone to complications such as tibial fracture, osteomyelitis, and skin necrosis (36, 37). To mitigate these risks, we implemented smaller incisions and a two-segment osteotomy, which is less traumatic. Our study showed successful ulcer healing in all patients without any complications. The external fixation can be removed once the bone cortexes have returned to their original positions with-out waiting for the fracture to heal. Patients wore the external fixation device for only 26–38 days (mean, 32 ± 3.61 days), which was a much shorter time than early chosen (15). However, it is recommended that proper weight-bearing activities be performed while wearing the orthosis for protection.

In group A, the results of the CTA for the lower limbs one year after surgery showed that the main arteries of the lower limbs were significantly thickened, with a large amount of neovascularization and vascular network formation. The comparison of VAS scores, 2-PD, and ABI at one year after surgery revealed statistically significant differences between groups A and B. These suggest that TTT surgery may delay the occurrence of vascular restenosis, promote microcirculation regeneration, enhance the long-term efficacy of PTA surgery, and improve the sensory function of the lower limbs. Therefore, using the TTT technique for treatment after PTA surgery is beneficial. How-ever, this study has several limitations, including its single-center design and small sample size. Further large-scale multicenter clinical research is imperative.

## 5 Conclusion

The sequential three-stage approach based on the ISWT mode is a reliable method for treating clinical stage 4 DFUs. It can rapidly control infection, achieve early wound healing, reduce hospital stay, increase limb salvage rate, and improve patients' quality of life. The combined use of TTT surgery at 3 stage treatment may enhance the sensory function of the lower limbs, reduce the mortality rate, improve the long-term results of PTA surgery, and potentially delay the occurrence of restenosis and occlusion.

## Data Availability

The original contributions presented in the study are included in the article/Supplementary Material, further inquiries can be directed to the corresponding authors.
