# Supplementary material for: A three-stage sequential surgical approach to a more efficient management of clinical stage 4 diabetic foot ulcers
Source: Front Surg. 2025 Nov 27;12:1696424. doi: 10.3389/fsurg.2025.1696424 (PMC12696158; doi:10.3389/fsurg.2025.1696424)
Supplement: Supplementary file 1 [file Table1.docx]

**Supplementary Table 1. Ulcers and amputations data of patients**

| Cases | Age (year) | Gender | Ulcer location | Ulcer area (cm^2^) | Extent of amputation |
| --- | --- | --- | --- | --- | --- |
| **Group A** |  |  |  |  |  |
| Case 1 | 42 | Male | dorsum pedis | 96 | none |
| Case 2 | 57 | Male | planta pedis | 82 | none |
| Case 3 | 58 | Female | dorsum pedis, medial border of foot | 76 | none |
| Case 4 | 49 | Male | dorsum pedis, planta pedis | 126 | The middle part of the 3-4 metatarsal and the proximal part of the 5 metatarsal |
| Case 5 | 65 | Female | dorsum pedis | 97 | The distal part of the 1-4 metatarsal |
| Case 6 | 62 | Male | dorsum pedis, medial border of foot | 87 | none |
| Case 7 | 57 | Female | dorsum pedis, medial border of foot | 68 | none |
| Case 8 | 49 | Male | dorsum pedis | 75 | none |
| Case 9 | 53 | Male | dorsum pedis, planta pedis | 98 | The distal part of the 2-3 metatarsal |
| Case 10 | 61 | Female | dorsum pedis | 88 | The middle part of the 2-4 metatarsal |
| Case 11 | 67 | Male | dorsum pedis, lateral border of foot | 96 | none |
| **Group B** |  |  |  |  |  |
| Case 1 | 68 | Male | dorsum pedis | 81 | none |
| Case 2 | 67 | Female | lateral border of foot | 74 | The distal part of the 5 metatarsal |
| Case 3 | 44 | Male | planta pedis | 94 | none |
| Case 4 | 56 | Female | dorsum pedis, planta pedis | 128 | none |
| Case 5 | 58 | Male | dorsum pedis, medial border of foot | 126 | The proximal part of the 1-5 metatarsal |
| Case 6 | 49 | Female | lateral border of foot | 62 | none |
| Case 7 | 62 | Male | lateral border of foot | 63 | none |
| Case 8 | 57 | Female | dorsum pedis | 78 | none |
| Case 9 | 48 | Male | dorsum pedis | 52 | none |
| Case 10 | 55 | Male | planta pedis, medial and lateral border of foot | 130 | The middle part of the 2-5 metatarsal |
| Case 11 | 64 | Female | dorsum pedis, lateral border of foot | 98 | none |
| Case 12 | 54 | Male | dorsum pedis, medial border of foot | 90 | none |
